# Genome-Wide Identification of the *SWEET* Gene Family in Peanut and Prediction of Candidate *AhSWEET* Genes Associated with Seed Protein Accumulation

**DOI:** 10.3390/ijms27156924

**Published:** 2026-08-01

**Authors:** Lina He, Pengyu Qu, Xiaona Li, Huanhuan Zhao, Lulu Xue, Yuanjin Fang, Liuyang Fu, Xiaobo Wang, Suoyi Han, Li Qin, Xia Shi, Xiaodong Dai, Lei Shi, Xinyou Zhang

**Affiliations:** 1 School of Life Sciences, Zhengzhou University, Zhengzhou 450001, China; he19031@foxmail.com (L.H.); 17837860522@163.com (P.Q.); fuly@zzu.edu.cn (L.F.); 2 Institute of Crop Molecular Breeding, Henan Academy of Agricultural Sciences/Key Laboratory of Oil Crops in Huang-Huai-Hai Plains, Ministry of Agriculture and Rural Affairs/Henan Provincial Key Laboratory for Oil Crops Improvement/National and Provincial Joint Engineering Laboratory for Peanut Genetic Improvement, Zhengzhou 450002, China; li12568xiaona@163.com (X.L.); z2023huan@163.com (H.Z.); luluxue9331@163.com (L.X.); sunfloweryuanjin@163.com (Y.F.); biowangxb@163.com (X.W.); suoyi_han@126.com (S.H.); qinli1018@126.com (L.Q.); mrshi0614@126.com (X.S.); leis100@163.com (L.S.); 3 The Shennong Laboratory, Zhengzhou 450002, China; 4 National Invocation Center for Bio-Breeding Industry, Zhengzhou 450002, China

**Keywords:** peanut, *SWEET*, sugar transport, gene family, seed quality

## Abstract

The cultivated peanut (*Arachis hypogaea* L.) is an important oilseed and economic crop worldwide, with seed protein content being a key target for quality improvement. The Sugars Will Eventually be Exported Transporter (SWEET) gene family encodes sugar transporters that play crucial roles in plant growth and development, stress tolerance, pathogen interactions, and seed filling. In this study, 43 *AhSWEET* genes were identified in the cultivated peanut genome and classified into four phylogenetic clades. Members within the same clade generally show similar exon-intron structures and conserved motif compositions, suggesting evolutionary conservation within subgroups. Promoter cis-regulatory elements analysis indicated that *AhSWEET* genes contain multiple elements associated with light response, hormone signaling, stress response, and growth and development, implying their potential involvement in diverse biological processes. Subcellular localization predictions indicated that most AhSWEET proteins are likely localized to the plasma membrane, consistent with their putative roles in transmembrane sugar transport. This was further supported by transient expression analysis of selected AhSWEET-eGFP fusion proteins in *Nicotiana benthamiana* leaves. Comparative phylogenetic and synteny analyses reveal that *AhSWEET5*, *AhSWEET21*, *AhSWEET27*, and *AhSWEET43* are closely related to soybean *GmSWEET10a* and *GmSWEET10b*, which are known to regulate seed size and storage-compound accumulation. Transcriptome data and quantitative reverse transcriptase PCR analysis showed that these candidate genes are preferentially expressed in seed-related tissues, particularly the testa and embryo, suggesting possible sugar allocation during peanut seed development. Overall, this study provides a systematic characterization of the *AhSWEET* gene family and identifies several candidate genes for future functional studies aimed at improving peanut seed quality, including protein accumulation.

## 1. Introduction

Oilseeds are increasingly recognized as promising sources of plant-based food; their composition promotes interactions between lipids and proteins, thereby positively influencing protein digestion, absorption, and metabolism [1]. Peanut (*Arachis hypogaea* L.), belonging to the Fabaceae or Leguminosae family, is a widely cultivated economic crop worldwide. Peanut seeds typically contain 22% to 28% protein [2,3]. Peanut protein has attracted increasing attention as a high-quality plant-based protein source due to its low level of antinutritional factors, high digestibility, and potential health advantages compared with animal protein-based diets [4]. With the development of the processing industry, peanut protein has become the third-largest source of plant proteins in the human diet, accounting for around 11% of the global protein supply [5]. Therefore, the cultivation of high-protein peanut varieties has become one of the important directions in peanut breeding and quality improvement [6].

Sucrose is a key carbon source for the synthesis of seed storage compounds, including lipids, proteins, and carbohydrates. Previous studies have demonstrated that sugar transporters play critical roles in the formation and regulation of seed storage substance composition [7,8]. Intercellular sugar transport requires the coordinated action of multiple transporter families, including Monosaccharide Transporters (MSTs), Sucrose Transporters (SUTs), and Sugars Will Eventually be Exported Transporters (SWEETs) [9], among which SWEETs perform essential functions in sugar efflux from cells [10]. The *SWEET* gene family is an extensively studied class of sugar transporters that can facilitate sugar movement across membranes along concentration gradients [11]. SWEET proteins are typically composed of seven transmembrane helices (TMs) and contain two MtN3/saliva (MtN3_slv) domains [12]. In plants, the *SWEET* gene family plays important roles in regulating sugar distribution, growth, and development, and stress responses [13,14]. Consequently, *SWEET* genes have become important targets in crop genetic improvement.

The transfer of sugars helps balance energy supply and modulate osmotic pressure, thereby regulating plant growth and development, as well as responses to biotic and abiotic stresses [15]. In *Arabidopsis thaliana*, the expression levels of *AtSWEET11* and *AtSWEET12* significantly increase under drought conditions, which enhances root growth and thereby improves drought tolerance [16]. Overexpression of *AtSWEET4* increases glucose and fructose levels in plants and significantly enhances cold tolerance [17]. Studies in other species have yielded similar conclusions: *StSWEET1g* in *Solanum tuberosum* activates the jasmonic acid signaling pathway, thereby broadly inhibiting infection by various RNA viruses [18].Transient overexpression of *BpSWEET1c* in *Betula platyphylla* Sukaczev enhances cold tolerance [19]. Growing evidence indicates that *SWEET* genes play important roles in plant stress responses by regulating sugar transport and redistribution.

Sugar allocation influences embryonic development and is closely associated with oil and protein biosynthesis [8]. In *Oryza sativa*, *OsSWEET11* plays an indispensable role during early grain filling; knocking out *OsSWEET11* impairs grain filling, directly leading to reduced growth, grain weight, and grain-setting rate [20,21]. Changes in the *SWEET* expression frequently influence the expression of other genes. For example, knocking out *OsSWEET7c* significantly reduces the expression of grain-filling-related genes, including *GIF1*, *OsNF-YB1*, and *OsPK3*, resulting in decreased grain width, grain thickness, and thousand-grain weight, suggesting that *SWEET* genes may coordinate with these genes during seed development [22]. Similarly, *GhSWEET42* in *Gossypium hirsutum* has been reported to positively regulate seed size and oil content [23]. These studies demonstrate that sugar transport between maternal and filial seed tissues is crucial for carbon allocation, embryo development, and seed yield.

In leguminous crops closely related to peanut, research on SWEET function has also yielded important advances. Simultaneous knockout of *GmSWEET15a*/*b* in *Glycine max* leads to greatly reduced embryo sugar content, resulting in severe seed abortion [24]. *GmSWEET12*/*21* is upregulated by drought, transiently increasing sugar transport to seeds [25]. *GmSWEET20*, *GmSWEET29*, and *GmSWEET34* are believed to mediate seed protein content [26,27]. Among these genes, *GmSWEET10a* and *GmSWEET10b* are considered among the most influential genes identified to date in soybean domestication for yield and quality traits [28]. Both genes are expressed in the thick-walled parenchyma of the seed coat, where they drive the transport of sucrose and hexoses from the seed coat to the embryo and synergistically regulate soybean seed size, oil content, and protein content [8]. Simultaneous knockout of both *GmSWEET10a* and *GmSWEET10b* reduces soybean oil content by 40.7% while increasing protein content by 32.1% [29]. Multiple studies have also found that genes such as *GmSop20*, *GmDt1*, and *GmST05* likely regulate seed yield and quality by influencing *GmSWEET10a*/*b* [30,31,32]. At the soybean seed-storage stage, sucrose, as the major carbon source, is hydrolyzed and exported into the cell-wall space, and then imported into the embryo by sugar transporters. Both oil and protein originate from the production of glycolysis-derived pyruvate, and there is competition for substrates between oil and protein production. Altered expression of sugar transporters may decrease the precursor of acetyl-CoA available for oil biosynthesis, thereby suppressing lipid accumulation. This metabolic shift could redirect glycolytic intermediates toward amino acid biosynthesis through the phosphoenolpyruvate carboxylases (PEPCs) pathway, potentially promoting protein accumulation [33,34,35]. These findings reveal a central role of SWEET-mediated sugar transport in seed storage compound accumulation in leguminous crops and provide important insights for editing *AhSWEET* genes to enhance protein content in peanuts.

In this study, we comprehensively identified SWEETs in *A. hypogaea* and analyzed their phylogenetic relationships, chromosomal distribution, syntenic relationships, selection pressure, conserved motifs, gene structures, promoter cis-regulatory elements (CREs), expression profiles, and subcellular localization. These analyses provide a systematic understanding of the structural characteristics, evolutionary relationships, and potential functional diversification of *AhSWEET* genes. In particular, comparative analysis with soybean *GmSWEET10a*/*b* allowed us to identify candidate *AhSWEET* genes that may participate in sugar allocation during peanut seed development. This study provides a foundation for further functional characterization of *AhSWEET* genes and for future molecular breeding or gene-editing efforts aimed at improving peanut seed quality.

## 2. Results

### 2.1. Characterization of the AhSWEET Gene Family in Peanuts

To investigate the evolutionary patterns of the *SWEET* gene family in plants, we analyzed seven representative species, including the monocotyledonous model maize, the dicotyledonous model plant *A. thaliana*, and five leguminous species, including *A. hypogaea*, *G. max*, *Medicago truncatula*, *Arachis duranensis*, and *Arachis ipaensis*.

Using the amino acid sequences of 17 SWEETs previously reported in *A. thaliana* as query sequences, we performed domain searches against the protein databases of each species using the Hidden Markov Model (HMM) profile PF03083, followed by BLASTp (version 2.17.0) homology searches. The candidate SWEET proteins were further confirmed using NCBI CD-Search (version 3.21) and SMART (version 10) to verify the presence of conserved MtN3_slv domains in *A. hypogaea* cv. YZ9102; 43 putative *SWEET* genes were identified. In comparison, 25 and 28 *SWEET* genes were identified in its diploid progenitors *A. duranensis* and *A. ipaensis*, respectively. The number of *SWEET* genes in other representative species was 20 in *Zea mays*, 17 in *A. thaliana*, 27 in *M. truncatula*, and 48 in *G. max*.

Based on their chromosomal distribution in *A. hypogaea* cv. YZ9102, the 43 *SWEET* genes were designated *AhSWEET1* to *AhSWEET43*. These genes encode proteins ranging from 86 to 312 amino acids in length, with predicted molecular weights (MW) from 9.83 to 34.38 kDa and theoretical isoelectric points (pI) from 4.55 to 9.71. Each protein contains one to two MtN3_slv domains and two to seven TMs. Most AhSWEET proteins possess seven predicted TMs, which is consistent with the typical structure of plant SWEET transporters. However, AhSWEET38 and AhSWEET42 contain only two predicted TMs, suggesting that these two members may represent truncated proteins, incomplete annotations, or functionally divergent SWEET-like proteins. Subcellular localization predictions indicated that most AhSWEET proteins are localized to the plasma membrane, whereas several members were predicted to be in the chloroplast (Table 1).

### 2.2. Phylogenetic Analysis of AhSWEET Proteins

**Figure 1:**
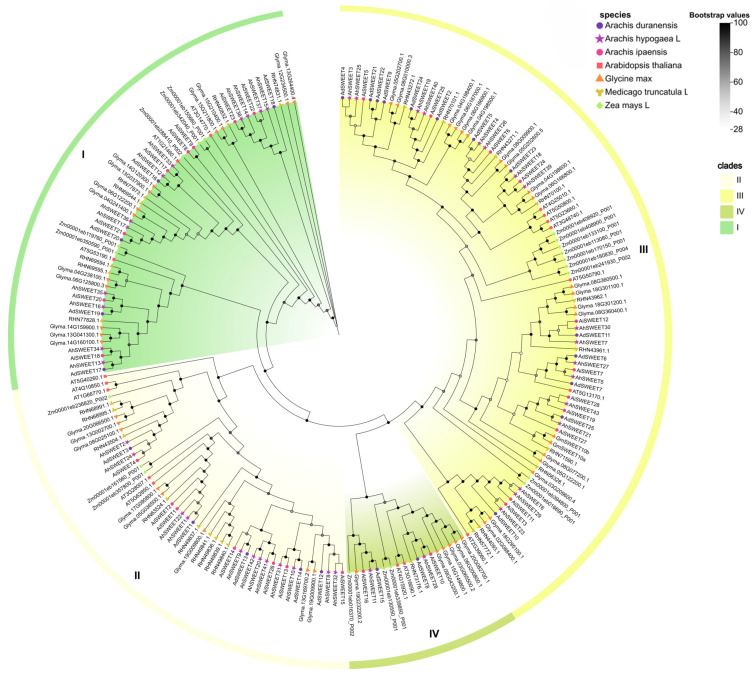
Phylogenetic relationships of 208 SWEETs from seven species. SWEET family members were categorized into four clades, each represented by a different color. Different shapes indicate different plant species (*Zea mays*, *Arabidopsis thaliana*, *Arachis hypogaea*, *Arachis duranensis*, *Arachis ipaensis*, *Medicago truncatula*, and *Glycine max*). Bootstrap analysis was performed with 1000 replicates to assess branch support. The scale and color bar indicate bootstrap confidence values ranging from 0.5 to 1.

To investigate the evolutionary relationships among SWEET proteins, a phylogenetic tree was constructed using 208 identified SWEET proteins from the seven selected species by the Maximum Likelihood (ML) method in MEGA 11. Based on the clustering pattern, these SWEET proteins were divided into four clades: Clade I, Clade II, Clade III, and Clade IV. Among them, Clade III contained the largest number of members (88), followed by Clade I (53), Clade II (49), and Clade IV (18). Several peanut SWEET proteins were clustered with soybean GmSWEET10a and GmSWEET10b in Clade III. In particular, AhSWEET21, AhSWEET43, AhSWEET5, AhSWEET27, AhSWEET7, and AhSWEET30 were located in the same phylogenetic group as GmSWEET10a/b (Figure 1). Because GmSWEET10a and GmSWEET10b have been implicated in storage-compound accumulation in soybean, these *AhSWEET* members may be candidate genes involved in similar biological processes in peanuts. However, their specific functions require further experimental validation.

As shown in Figure 1, the combined number of *SWEET* genes in the two diploid progenitors *A. duranensis* and *A. ipaensis* was higher than that in cultivated allotetraploid peanut. This difference may reflect gene loss or fractionation after polyploidization [36]. However, possible effects of genome assembly quality, annotation differences, and cultivar-specific variation should also be considered. Therefore, this result suggests, but does not by itself prove, dynamic changes in the *SWEET* gene family during peanut genome evolution.

### 2.3. Chromosomal Localization, Collinearity Analysis, and Selection Pressure Analysis of AhSWEET Genes

**Figure 2:**
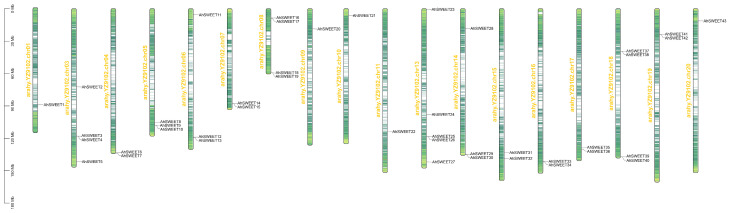
Chromosome distribution of *AhSWEET* genes in the *Arachis hypogaea* genome. The physical locations of 43 *AhSWEET* genes were mapped onto 18 chromosomes of *A. hypogaea* cv. YZ9102. Chromosomes are shown in vertical orientation and labelled with their respective names. *AhSWEET* gene names are indicated in black, and their chromosomal positions are marked accordingly. Gene density is depicted in a color gradient, with green indicating regions of higher gene density and white indicating regions of lower gene density.

To systematically analyze the chromosomal distribution of *AhSWEET* genes, we mapped the physical positions of the 43 *AhSWEET* genes onto the chromosomes of cultivated peanut. The results showed that the 43 *AhSWEET* genes were unevenly distributed across 18 chromosomes. No *AhSWEET* genes were identified on chromosomes 02 and 12, whereas the remaining chromosomes contained one or more *AhSWEET* genes. Most *AhSWEET* genes were located near telomeric regions (Figure 2). This distribution is consistent with the general genomic features of peanut, in which many genes are enriched in chromosome terminal regions [37,38].

**Figure 3:**
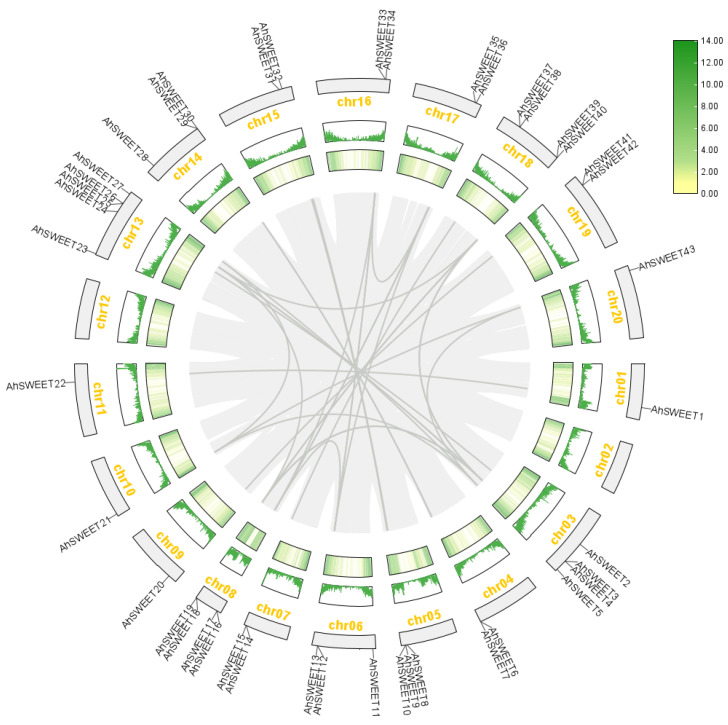
Intraspecific syntenic relationships of *AhSWEET* genes in the *Arachis hypogaea* genome. The circular plot shows syntenic relationships among *AhSWEET* genes across the 20 chromosomes of *A. hypogaea* (AABB genome). Gene locations are labeled on the outer ring, with green histograms and heatmaps indicating gene density. Gray connecting lines within the circle indicate 31 syntenic gene pairs, highlighting conserved segmental duplications primarily between the homologous regions of the A and B subgenomes.

Because cultivated peanut is an allotetraploid species, many *AhSWEET* genes exhibited conserved collinear relationships between homologous regions of the A and B subgenomes. Gene family expansion can occur through whole-genome duplication (WGD), tandem duplication, and segmental duplication [39,40]. Based on intraspecific colinearity analysis, 31 syntenic *AhSWEET* gene pairs were identified (Figure 3). No tandem duplication events were detected, indicating that WGD or segmental duplication was the major contributor to the expansion and retention of the *AhSWEET* gene family in cultivated peanut.

To evaluate selective pressure acting on duplicated *AhSWEET* gene pairs, nonsynonymous-to-synonymous substitution rate ratios (Ka/Ks) were calculated for the intraspecific syntenic pairs [41]. Among the 31 identified gene pairs, most had Ka/Ks ratios lower than 1 (Table 2), indicating that these duplicated genes have generally experienced purifying selection during evolution. This suggests that many *AhSWEET* genes have retained conserved functions after duplication. One gene pair, *AhSWEET12*/*AhSWEET33*, showed a Ka/Ks ratio greater than 1, suggesting possible selection or functional divergence.

**Figure 4:**
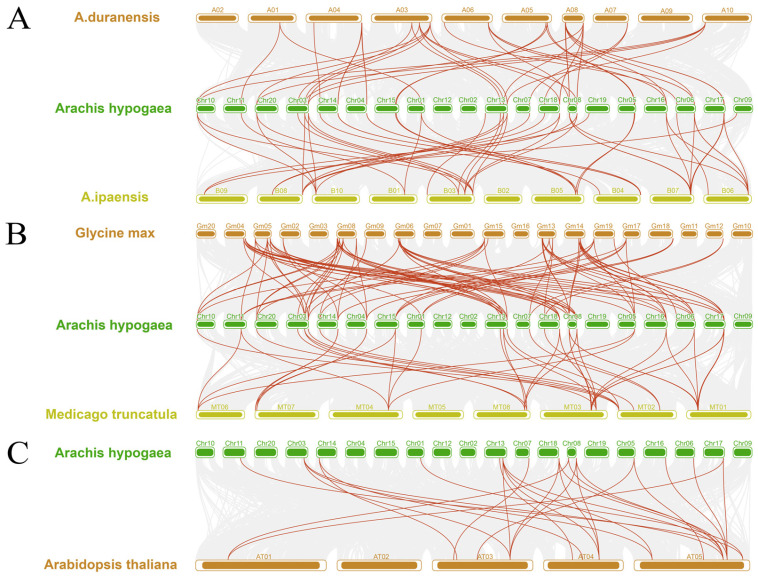
Interspecific syntenic relationships of *AhSWEET* genes between *Arachis hypogaea* and five other plant species. Red lines indicate conserved syntenic gene pairs. (**A**) Collinearity between *A. hypogaea* and its wild diploid progenitors *A. duranensis* (AA genome) and *A. ipaensis* (BB genome). (**B**) Collinearity between *A. hypogaea* and the related leguminous species *G. max* and *M. sativa*. (**C**) Collinearity between *A. hypogaea* and the model species *A. thaliana*.

To further investigate the evolutionary relationships of *AhSWEET* genes, interspecific synteny analyses were performed between *A. hypogaea* and five representative species, including its two progenitors *A. duranensis* and *A. ipaensis*, two leguminous species *G. max* and *M. truncatula*, and the model dicotyledonous plant *A. thaliana*. The analysis identified 46 syntenic gene pairs between *A. hypogaea* and *A. duranensis* and 50 pairs between *A. hypogaea* and *A. ipaensis*, reflecting their close evolutionary relationship. In addition, 44 and 100 syntenic gene pairs were identified between *A. hypogaea* and *M. truncatula* and *G. max*, respectively, whereas only 27 syntenic pairs were identified between *A. hypogaea* and *A. thaliana* (Figure 4). These results are consistent with the phylogenetic relationships among these species. All interspecific syntenic gene pairs had Ka/Ks ratios lower than 1 (Table S1), suggesting that these genes have generally been conserved under purifying selection.

Among the syntenic gene pairs between *A. hypogaea* and *G. max*, one *AhSWEET* was often collinear with multiple *GmSWEET* genes. This may be related to two WGD events that occurred during soybean evolution. Due to the relatively slow process of post-polyploid diploidization, most genes are present in multiple copies across the soybean genome, resulting in more syntenic gene pairs compared to the other species [42]. Notably, *AhSWEET21*, *AhSWEET43*, *AhSWEET5*, and *AhSWEET27* showed syntenic relationships with *GmSWEET10a* and *GmSWEET10b*, suggesting that these four genes may represent strong peanut candidate counterparts of soybean *GmSWEET10a*/*b*. In contrast, *AhSWEET7* and *AhSWEET30* clustered phylogenetically with *GmSWEET10a*/*b* but did not show clear syntenic relationships, suggesting that their functions may have diverged.

### 2.4. Conserved Motif and Domain Analysis of AhSWEET

**Figure 5:**
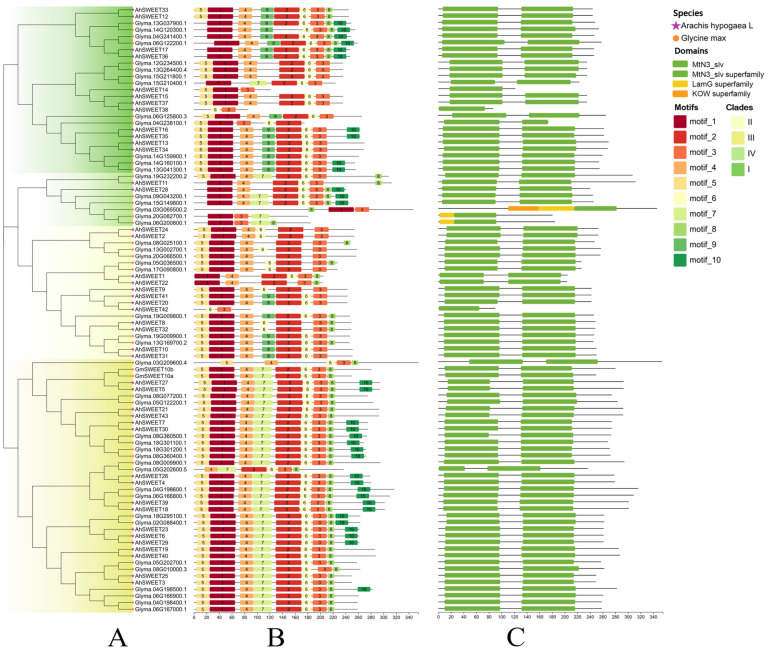
Conserved motifs and protein domain architectures in *SWEET* gene families in *Glycine max* and *Arachis hypogaea*. (**A**) Maximum Likelihood (ML) phylogenetic tree of *G.max* and *A. hypogaea*, with clades distinguished by different colors. (**B**) Conserved motif composition of all SWEETs. Ten motifs (motif_1 to motif_10) are represented by colored boxes. (**C**) Predicted protein domain structures of *GmSWEETs* and *AhSWEETs*. Green boxes indicate the MtN3_slv domain, and additional domains (e.g., LamG superfamily and KOW superfamily) are labeled accordingly.

To further investigate potential functional divergence among AhSWEET proteins, we used the MEME Suite and NCBI CD-Search tools to analyze the conserved motifs and protein domains in *G. max* and *A. hypogaea*. A total of ten distinct conserved motifs were identified across all *SWEET* genes. The number of motifs in individual *AhSWEET* genes ranged from 2 to 9, with *AhSWEET38* and *AhSWEET42* containing the fewest (two), while *AhSWEET4*, *AhSWEET5*, *AhSWEET6*, *AhSWEET7*, *AhSWEET18*, *AhSWEET23*, *AhSWEET26*, *AhSWEET27*, *AhSWEET29*, *AhSWEET30*, and *AhSWEET39* contain the most (nine) (Figure 5). Members within the same clade typically shared similar conserved motif compositions. *AhSWEET21* and *AhSWEET43* in clade III exhibit motif patterns identical to *GmSWEET10a* and *GmSWEET10b*, implying they likely maintain analogous biochemical functions. However, *AhSWEET26*, *AhSWEET4*, *AhSWEET39*, *AhSWEET18*, *AhSWEET23*, *AhSWEET6*, *AhSWEET29*, *AhSWEET7*, *AhSWEET30*, *AhSWEET5*, and *AhSWEET27* contain motif 10 in contrast to other clade III members, suggesting possible functional divergence.

Differences in motif composition may reflect structural diversification among AhSWEET proteins. Notably, certain motifs are absent in particular clades; for example, motif 7 is absent in clade II, while motif 9 is absent in clades III and IV. The specific motifs across clades may contribute to functional diversity among *AhSWEET* genes. Their biological significance remains to be experimentally verified.

### 2.5. Gene Structure and Cis-Regulatory Elements Analysis of the AhSWEET Gene Family

**Figure 6:**
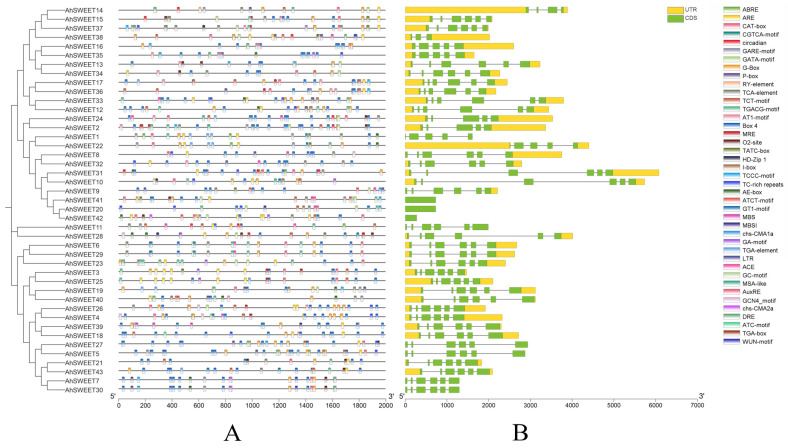
Promoter cis-regulatory element (CRE) distribution and gene structure of *AhSWEET* genes. (**A**) Predicted CRE within the 2000 bp upstream promoter regions of *AhSWEET* genes. Different colored symbols represent distinct functional categories of regulatory elements. (**B**) Gene structure analysis of *AhSWEET* genes. Green boxes represent coding sequences (CDSs), and yellow boxes represent untranslated regions (UTRs). The gene models highlight variations in exon–intron organizations among gene family members.

Using the PlantCARE database, we performed a CRE analysis of the promoter sequences within the 2000 bp upstream promoter regions of *AhSWEET* genes. The results showed that a total of 1056 CREs were identified in the promoter regions of the 43 *AhSWEET* genes (Table S2). Based on the different biological processes, these CREs can be classified into five categories: light response (556), hormone signaling (292), development (77), stress tolerance (100), and other elements (31) (Figure 6). Among these, light-responsive elements were the most abundant and widely distributed across the promoters, including the Box 4 element, ATCT-motif element, MRE element, GATA-motif element, and so on, suggesting the expression may be light-regulated. In addition to light-responsive elements, various elements associated with growth and development regulation were identified. Twenty-nine genes, including *AhSWEET5*, *AhSWEET7*, *AhSWEET21*, *AhSWEET27*, *AhSWEET30*, and *AhSWEET43*, contain the CAT-box element, which participates in meristem expression, suggesting their potential role in meristematic and organ development. The presence of seed-specific elements such as the GCN4_motif and RY-element in *AhSWEET14*, *AhSWEET4*, *AhSWEET8*, *AhSWEET26*, and *AhSWEET28* implies they may participate in seed development.

Every *AhSWEET* promoter harbored at least two hormone signaling elements, including gibberellin (TATC-box, GARE-motif), methyl jasmonate (CGTCA-motif, TGACG-motif), salicylic acid (TCA-element), abscisic acid (ABRE), and auxin (TGA-element, AuxRR-core), suggesting the possible involvement of *AhSWEET* genes in multiple hormonal signaling pathways. Furthermore, the promoters of twelve *AhSWEET* genes contained the drought-inducible element MBS, five genes carried the cold-responsive element LTR, and 16 genes carried the defense and stress-responsive TC-rich repeats (TC-rich repeats), suggesting that *AhSWEET* genes may play an important role in responses to abiotic stresses.

To further explore the diversity of *AhSWEET* genes structures, we analyzed their exon-intron structures based on genomic sequences. The results revealed that every *AhSWEET* gene ranged from one to six exons, with most *AhSWEET* genes having 6 exons; however, *AhSWEET20*, *AhSWEET41*, and *AhSWEET42* contained only 1 exon (Figure 6). Combined analysis of phylogenetic trees and gene structure revealed that most *AhSWEET* genes located on the same clade exhibited gene structural similarities. The variation in the number of exons among different *AhSWEET* genes suggests functional diversity.

### 2.6. Expression Patterns of SWEET Genes in A. hypogaea

To investigate the expression profiles of the *AhSWEET* gene family members, we analyzed the transcriptomic data from 19 different tissues of the peanut cv. Shitouqi at various developmental stages [43]. *AhSWEET* genes showed different expression levels in all tissues, with some genes showing preferential expression in certain tissues (Figure S1). For example, *AhSWEET16*, *AhSWEET35*, *AhSWEET19*, and *AhSWEET40* showed high expression levels (FPKM > 10) [44] in Testa-II, suggesting these *AhSWEET* genes might play important roles in transmembrane sugar transport during seed development. *AhSWEET16*, *AhSWEET13*, *AhSWEET35*, *AhSWEET11*, *AhSWEET19*, and *AhSWEET40* showed high expression levels in root nodules (FPKM > 10), suggesting they may contribute to carbon source provision for bacteroids (Table S3).

**Figure 7:**
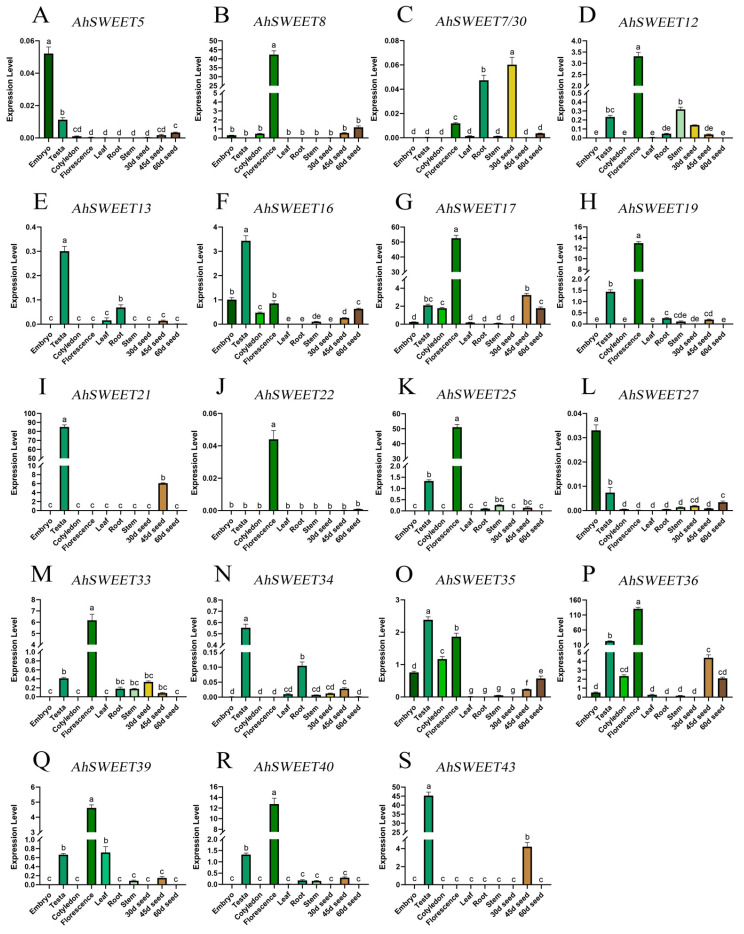
Tissue-specific expression profiles of *AhSWEET* genes in peanuts. (**A**–**S**) The relative expression levels of 20 *AhSWEET* genes were analyzed across ten different tissues, including Embryo, Testa, Cotyledon, Flower, Leaf, Root, Stem, 30d seeds, 45d seeds, and 60d seeds, using quantitative reverse transcriptase PCR (RT-qPCR). Expression data were normalized against the internal reference gene *AhADH3*. Error bars indicate the standard deviation of triplicate biological replicates. Different letters above the bars denote significant differences (*p* < 0.05) among tissues.

We selected 20 *AhSWEET* genes that exhibited some level of expression in developing peanut seeds, then performed quantitative reverse transcriptase PCR (RT-qPCR) to investigate the expression of *AhSWEET* genes in different tissues of cultivated peanut (Table S4). The results showed that 20 *AhSWEET* genes exhibited high expression levels in flowers, embryos, and testa (Figure 7), implying their potential roles in developmental stages. For example, *AhSWEET8*, *AhSWEET19*, *AhSWEET25*, and *AhSWEET36* are preferentially expressed in flowers, suggesting these genes might be involved in floral development. Although the expression levels of *AhSWEET13* and *AhSWEET34* were generally low, their expression in the testa was higher than in seeds at all developmental stages, suggesting that they may be involved in sucrose translocation to the embryo, which was important for seed dry matter accumulation. The expression of most *AhSWEET* genes was upregulated at 30 and 45 days after pollination, implying their potential function in seed development.

Phylogenetic analysis revealed that *AhSWEET7*, *AhSWEET30*, *AhSWEET5*, *AhSWEET21*, *AhSWEET27*, and *AhSWEET43* were closely related to soybean *GmSWEET10a* and *GmSWEET10b*. *GmSWEET10a*/*b* are specifically expressed in the seed coats, where they likely transport sugars from seed coats to embryos and genetically regulate seed size and composition [29]. *AhSWEET21* and *AhSWEET43* displayed high expression levels in the testa. Although *AhSWEET5* and *AhSWEET27* have generally low expression levels, their expression in the embryos and testa is significantly higher than in other tissues. These *AhSWEET* genes showed an expression pattern similar to the *GmSWEET* genes, suggesting that they may share similar functions, such as leading to the flux of more sugars into embryos from maternal tissues. In contrast, although AhSWEET7 and AhSWEET30 were located in the same phylogenetic group as GmSWEET10a/b, they were preferentially expressed in 30d seeds and roots but were barely expressed in the embryo and testa, suggesting that their functions may have diverged. Comparative analysis with soybean *GmSWEET10a*/*b* allowed us to identify candidate *AhSWEET* genes that may participate in sugar allocation during peanut seed development. Their specific functions require further experimental validation.

### 2.7. Subcellular Localization of SWEETs in Arachis hypogaea

**Figure 8:**
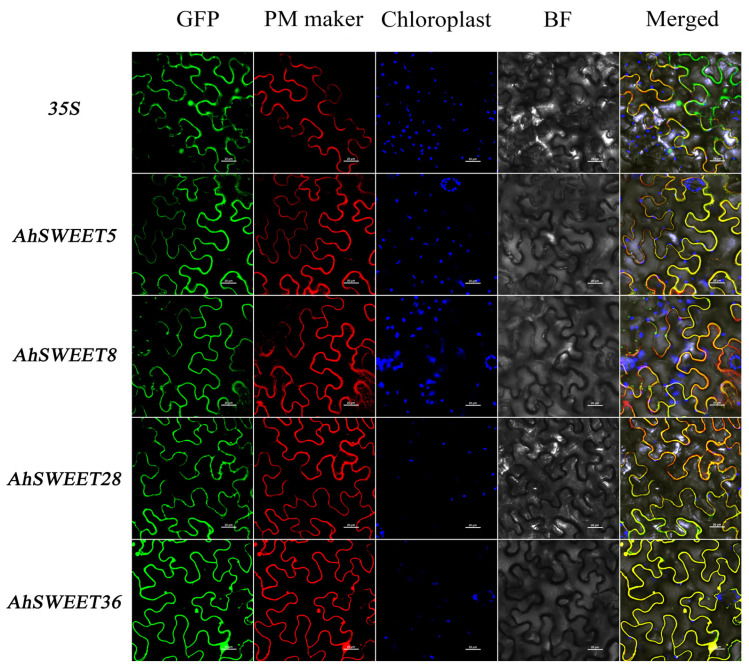
The subcellular localization of selected *AhSWEETs* in *Nicotiana benthamiana* epidermal cells. The transient expression of eGFP-tagged AhSWEET5, AhSWEET8, AhSWEET28, and AhSWEET36 fusion proteins was observed in *N. benthamiana* leaves using confocal laser scanning microscopy. Positive control experiments were conducted using the 35S::eGFP construct. Columns from left to right show eGFP fluorescence, plasma membrane markers (red), chloroplast (blue), a bright-field image, and merged channels. Scale bars = 20 µm.

Subcellular localization is crucial for the precise functional annotation of proteins. To further explore the subcellular localization of AhSWEET proteins, we selected four genes, *AhSWEET36* (Clade I), *AhSWEET8* (Clade II), *AhSWEET5* (Clade III), and *AhSWEET28* (Clade IV), to construct four expression vectors. Then, we transiently expressed them in *Nicotiana benthamiana* leaves via *Agrobacterium*-mediated transformation. Confocal microscopy images showed that the four proteins exhibited strong co-localization with the plasma membrane marker protein (Figure 8). By contrast, the green fluorescence signal of the 35S::eGFP protein was observed throughout the cell, including cytoplasm, nucleus, and plasma membrane. The localization of AhSWEET5 to the plasma membrane mirrors that of GmSWEET10a in soybean [31], suggesting that they may share similar transmembrane transport functions. Overall, these results suggest that these AhSWEET proteins likely exert their potential biological functions at the plasma membrane, such as mediating transmembrane sugar transport and allocation, thereby regulating plant growth, development, and other physiological processes.

## 3. Discussion

The *SWEET* gene family, as a class of sugar transporters, regulates development and stress responses by mediating the transport and distribution of sugars. It has been systematically identified in other model plants, from A. thaliana to various crops such as *Hordeum vulgare* [45], *Glycine max* [46], and *Triticum aestivum* [47]. In this study, we identified 43, 25, and 28 *SWEET* genes in the genomes of *A. hypogaea* (AABB genome) and its diploid progenitors, *A. duranensis* (AA genome) and *A. ipaensis* (BB genome). The number of *AhSWEET* genes in the cultivated peanut is lower than the combined total of the diploid progenitors, likely due to the asymmetric evolution of the two subgenomes in *A. hypogaea* [48]. Some ancestral *SWEET* genes were lost or underwent fractionation after polyploidization [36], reflecting that the genes have undergone selective retention during evolution and may have contributed to environmental adaptation.

Based on the phylogenetic classification of the *A. thaliana* phylogenetic tree, AhSWEET proteins can be divided into four clades (Clade I–IV) [49]. Members of Clades I and II primarily transport glucose, fructose, and other sugars; Clade III preferentially transports sucrose; and Clade IV tends to transport fructose [50]. Genes clustered within the same clade exhibit similar motif compositions and domains. In this study, AhSWEET7, AhSWEET30, AhSWEET21, AhSWEET43, AhSWEET5, and AhSWEET27 were clustered with GmSWEET10a/10b, key genes validated in soybean that regulate seed size, oil content, and protein content. These genes are promising candidates for future functional studies. We found that most *AhSWEET* genes had six exons; this phenomenon was also reported in other species, such as rice [51] and wheat [52]. However, *AhSWEET20*, *AhSWEET41*, and *AhSWEET42* contain only one exon, possibly representing pseudogenized or functionally specialized members. Different *AhSWEET* genes exhibit variations in motif distribution and exon-intron structure, which may account for their divergent biological functions. This gene family expanded through WGD or segmental duplication events. Intraspecific and interspecific colinearity and Ka/Ks analyses of syntenic gene pairs indicate that the *AhSWEET* genes have undergone strong purifying selection during evolution, and their functions are highly conserved. Notably, the phenomenon in which a single *AhSWEET* gene is collinear with multiple *GmSWEET* genes in soybean may be related to the two WGD events that occurred in soybean [42]. In *A. hypogaea*, *AhSWEET21*, *AhSWEET43*, *AhSWEET5*, and *AhSWEET27* showed syntenic relationships with *GmSWEET10a* and *GmSWEET10b*, whereas *AhSWEET7* and *AhSWEET30* are not, suggesting that *AhSWEET21*, *AhSWEET43*, *AhSWEET5*, and *AhSWEET27* are putative candidate genes likely to share similar functions, whereas *AhSWEET7* and *AhSWEET30* may have diverged functions. Analysis of promoter CREs provides important evidence for the *AhSWEET* gene expression level. The 29 genes containing the meristem-associated element (CAT-box) may be expressed in embryonic development and organ formation. The presence of various hormone response elements and abiotic response stress elements suggests that *AhSWEET* genes may also be involved in stress responses, which is consistent with previously reported functions of *SWEET* genes [53,54,55].

Analysis of the expression patterns of the *AhSWEET* genes across different tissues provides insights into their potential biological functions. Transcriptomic expression profiling revealed that *AhSWEET* genes are preferentially expressed in the embryos, testa, flowers, root nodules, and cotyledons. This tissue-specific expression pattern is consistent with that of *SWEET* genes in other species. For instance, in maize, *ZmSWEET4c* is highly expressed in the basal endosperm transfer layer (BETL), where it enhances sugar import into the endosperm for starch synthesis [56]. In Arabidopsis, *AtSWEET8* and *AtSWEET13* have been demonstrated to provide sugars for pollen development, thereby ensuring normal male gametophyte development and pollen maturation [49]. In soybean, *GmSWEET39* is predominantly expressed in the parenchyma and integument of the seed coat, where it likely regulates oil and protein accumulation by modulating sugar delivery from the maternal seed coat to the filial embryo [57]. Additionally, *GmSWEET3c* is specifically activated during rhizobial infection, providing energy for nodulation [58]. Collectively, these findings indicate that these *SWEET* genes participate in diverse physiological processes, including seed dry matter accumulation, reproductive development, and nitrogen fixation through distinct sugar allocation strategies in different tissues.

RT-qPCR analysis was further performed to examine the expression patterns of *AhSWEET* genes, which suggested their potential functions in diverse physiological processes. For example, *AhSWEET8*, *AhSWEET19*, *AhSWEET25*, and *AhSWEET36* showed elevated expression in flowers, suggesting potential roles in flower development. *AhSWEET7*/*30* showed the highest expression levels in 30d seeds, suggesting their possible involvement in early seed development. *AhSWEET13* and *AhSWEET34* displayed relatively higher expression in the testa and roots, implying their potential participation in stress responses in roots. Notably, *AhSWEET5*, *AhSWEET27*, *AhSWEET21*, and *AhSWEET43* showed an expression pattern similar to *GmSWEET10a*/*b*, suggesting that they may share similar functions. Although *AhSWEET7* and *AhSWEET30* are closely related to *GmSWEET10a*/*b*, their expression patterns differ, suggesting that they may have evolved distinct biological functions.

Subcellular localization is one of the key factors determining protein function. Current research has confirmed that most of the identified SWEETs are localized to the plasma membrane [7,59], while a minority are targeted to the vacuolar membrane [60]. In this study, expression vectors were constructed by fusing the coding sequences of *AhSWEET8*, *AhSWEET28*, *AhSWEET36*, and *AhSWEET5* with an enhanced green fluorescent protein (eGFP) reporter gene. The results showed that the eGFP signals of all four AhSWEETs were highly co-localized with the plasma membrane marker protein. This is consistent with the hypothesis that they function as transmembrane sugar transporters [61]. Notably, AhSWEET28 was predicted to localize to the chloroplast (Table 1), whereas it was experimentally shown to localize to the plasma membrane. In silico localisation predictions based on N-terminal features for signal sequence detection and cleavage site identification are not always accurate [62], and thus experimental evidence is considered more reliable.

These findings expand our understanding of the crucial role of *SWEET* genes in peanuts. Based on their phylogenetic and synteny analyses and expression profiles, we identified four candidate AhSWEETs closely related to soybean GmSWEET10a and GmSWEET10b, which are known to regulate seed size and storage-compound accumulation, providing a theoretical foundation for future functional studies.

## 4. Materials and Methods

### 4.1. Identification and Analysis of the SWEET Gene Members

Genomic and annotation data for *Arachis hypogaea* cv. YZ9102 were obtained from the Figshare website (https://doi.org/10.6084/m9.figshare.26551309.v1, accessed on 22 November 2025) [63]. We obtained genomic data for the two wild diploid progenitors, *Arachis duranensis* (v1.SWBf) and *Arachis ipaensis* (v1.bxj8), from the PeanutBase database (https://www.peanutbase.org/, accessed on 1 December 2025). The genome sequence of *Glycine max* (v4.1) was retrieved from the SoyBase database (https://www.soybase.org/, accessed on 1 December 2025). We sourced additional reference genomes—including *Medicago truncatula* (v5.0), *Zea mays* (v5.0), and *Arabidopsis thaliana* (TAIR10)—from the Ensembl Plants database (https://plants.ensembl.org/index.html, accessed on 10 December 2025). We obtained the hidden Markov model (HMM) profile PF03083, corresponding to the MtN3_slv domains characteristic of the *SWEET* gene family, from the Pfam database (http://pfam.xfam.org/, accessed on 1 January 2026) [64,65]. Candidate sequences were retrieved from the protein databases of all selected species using the Simple HMM Search tool in TBtools (v2.441), with PF03083 as the query profile (E-value < 1 × 10^−5^). Using the reported amino acid sequences of *A. thaliana* SWEETs as reference sequences, a BLASTp (version 2.17.0) search was performed against the protein databases of target species. The resulting candidate gene sequences were submitted to SMART (http://smart.embl.de/, accessed on 10 January 2026) to identify conserved domains in *AhSWEET* genes. The candidate genes identified by both methods were selected, and sequences lacking the MtN3_slv domain were eliminated to improve the accuracy of member identification, ultimately yielding the *AhSWEET* gene family members [66]. The physicochemical properties of the *AhSWEETs* were analyzed using ExPASy (https://www.expasy.org/, accessed on 31 January 2026) [67], and their subcellular localization was predicted using Plant-mPLoc (http://www.csbio.sjtu.edu.cn/bioinf/plant-multi/, accessed on 31 January 2026) [68].

### 4.2. Multiple Sequence Alignment and Phylogenetic Analysis of SWEET Genes

We extracted the complete amino acid sequences of all *SWEET* gene family members from the seven plant species under study, performed multiple sequence alignment using DNAMAN 2.0, and constructed a phylogenetic tree using the Maximum Likelihood (ML) method in MEGA 11 software [69], with 1000 bootstrap replicates. The resulting tree was visualized using Chiplot (https://www.chiplot.online/, accessed 10 February 2026).

### 4.3. Chromosomal Localization, Gene Duplication, and Selective Pressure Analyses

Chromosomal localization of *AhSWEET* genes was performed based on the genome annotation data of *A. hypogaea* cv. YZ9102, and the location of *AhSWEET* genes on chromosomes was visualized using TBtools (v2.441). The MCScanX analysis implemented in TBTools was used to analyze intraspecific and interspecific duplications among peanuts and six other species [70], and the Ka/Ks Calculator module in TBTools was used to calculate the nonsynonymous-to-synonymous substitution rates (Ka/Ks) of duplicated *SWEET* gene pairs [41].

### 4.4. Conserved Domain and Motif Analysis of SWEET Genes

Conserved domains were identified using the NCBI Conserved Domain Database (CD-Search) (https://www.ncbi.nlm.nih.gov/Structure/bwrpsb/bwrpsb.cgi, accessed on 25 February 2026) [71], and the number of transmembrane domains was predicted using the DeepTMHMM tool (https://dtu.biolib.com/DeepTMHMM, accessed on 25 February 2026). MEME Suite under the 10-motif with default parameterization (http://meme-suite.org/tools/meme, accessed on 2 March 2026) was used to analyze conserved motifs in *AhSWEETs* [72].

### 4.5. Promoter Cis-Regulatory Element Prediction, Gene Structure, and Transcriptome Profile Analysis

Based on the genome sequence and GFF3 annotation file of *A. hypogaea* cv. YZ9102, the 2000 bp sequences upstream of the translational start sites were analyzed, and the types, numbers, and locations of CREs present in the sequence were predicted using PlantCARE (http://bioinformatics.psb.ugent.be/webtools/plantcare/html/, accessed on 3 March 2026) [73]. The intron and exon structures were mapped using the TBtools software (v2.441). The multi-tissue transcriptomic data of peanut cv. Shitouqi used in this study were obtained from Zhuang et al. [43]. The expression patterns of *AhSWEET* genes were analyzed using Prism (v8.0.2) software, and corresponding heatmaps were generated.

### 4.6. Plant Materials, RNA Isolation, and qRT-PCR

Total RNA was extracted from peanut cv. YH9326 using the MiniBEST Kit (Takara, Tokyo, Japan). cDNA was synthesized using the HiScript III All-in-one RT SuperMix Perfect for qPCR kit (Vazyme, Nanjing, China). After reverse transcription, quantitative reverse transcriptase PCR (RT-qPCR) was used to verify the expression patterns of *AhSWEET* genes in ten tissue types: the embryo, testa, cotyledon, root, stem, leaf, flower, 30d seeds, 45d seeds, and 60d seeds. The RT-qPCR primers are shown in Table S6. The reaction system and thermal cycling conditions were set according to the manufacturer’s instructions for the ChamQ Universal SYBR qPCR Master Mix (Vazyme, Nanjing, China). *AhADH3* was used as the internal reference gene. All reactions were conducted in triplicate, and relative gene expression levels were calculated using the 2^−∆Ct^ method [74].

### 4.7. Subcellular Localization of AhSWEETs

The coding sequences (CDS) of the cloned *AhSWEET5*, *AhSWEET8*, *AhSWEET28*, and *AhSWEET36* genes, excluding the stop codons (TAA), were fused into the pCAMBIA1300-eGFP vector via homologous recombination, generating the 35S::AhSWEET-eGFP fusion expression vectors. The recombinant constructs were transformed into *Agrobacterium tumefaciens* GV3101. The Agrobacterial suspensions harboring the target vectors were mixed with solutions carrying the plasma membrane marker at a 1:1 ratio, and the mixture was infiltrated into healthy leaves of *Nicotiana benthamiana* at the 4–6 leaf stage. After infiltration, the plants were incubated under low-light conditions for two days, and the distribution of eGFP fluorescence signals was subsequently observed using confocal laser scanning microscopy. The primer sequences for vector construction are documented in Supplementary Table S5.

## 5. Conclusions

In this study, we performed a genome-wide systematic identification and comparative analysis of the *SWEET* gene family in cultivated peanut. A total of 43 members of the *SWEET* gene family were identified and classified into four clades. All identified *AhSWEET* genes contained the conserved PF03083 domain and MtN3_slv domains.

The expansion of this family was driven by whole-genome duplication (WGD) and segmental duplication events. These genes have undergone strong purifying selection during evolution. *AhSWEET* genes within the same clade shared similar conserved motifs and exon–intron structures, suggesting highly conserved gene functions. Transient expression assays in *Nicotiana benthamiana* demonstrated that representative AhSWEETs were localized to the plasma membrane, consistent with their predicted identity as uniport transmembrane proteins.

RT-qPCR results revealed that *AhSWEET* genes were highly expressed in flowers, embryos, testa, and roots, suggesting that they may play diverse roles in physiological processes. Based on phylogenetic and expression profiling analyses, this study finds that AhSWEET5, AhSWEET21, AhSWEET27, and AhSWEET43 are closely related to soybean GmSWEET10a and GmSWEET10b and exhibit high homology and similar seed-related expression patterns. Collectively, this study provides a theoretical basis for further exploring the molecular mechanisms of sugar transport in peanut and identifies four candidate genes for improving peanut seed quality, including protein accumulation.

## Figures and Tables

**Table 1:** Physicochemical properties, transmembrane domains, and predicted subcellular localization of SWEET family proteins in *Arachis hypogaea* cv. YZ9102.

Chr	Gene ID	Gene Name	Protein Length (aa)	Molecular Weight (MW)/kDa	Isoelectric Point (pi)	Transmembrane Domains	Subcellular Location
Chr01	*g1836.t1*	*AhSWEET1*	204.00	23.32	8.88	5	Plasma membrane
Chr03	*g8630.t1*	*AhSWEET2*	253.00	27.92	9.37	6	Plasma membrane
Chr03	*g9017.t1*	*AhSWEET3*	249.00	28.29	9.71	7	Plasma membrane
Chr03	*g9019.t1*	*AhSWEET4*	279.00	31.48	8.99	7	Plasma membrane
Chr03	*g10485.t1*	*AhSWEET5*	293.00	32.90	8.97	7	Plasma membrane
Chr04	*g13930.t1*	*AhSWEET6*	261.00	29.73	6.99	7	Chloroplast
Chr04	*g14000.t1*	*AhSWEET7*	274.00	30.31	8.95	7	Plasma membrane
Chr05	*g16756.t1*	*AhSWEET8*	248.00	27.61	8.72	7	Plasma membrane
Chr05	*g16889.t1*	*AhSWEET9*	242.00	26.85	8.68	7	Plasma membrane
Chr05	*g16890.t1*	*AhSWEET10*	250.00	27.59	9.19	7	Plasma membrane
Chr06	*g18049.t1*	*AhSWEET11*	312.00	34.23	9.15	7	Plasma membrane
Chr06	*g20103.t1*	*AhSWEET12*	244.00	26.70	8.91	7	Plasma membrane
Chr06	*g20115.t1*	*AhSWEET13*	269.00	29.45	9.16	7	Plasma membrane
Chr07	*g23171.t1*	*AhSWEET14*	121.00	13.47	9.39	3	Plasma membrane
Chr07	*g23173.t1*	*AhSWEET15*	235.00	26.20	8.38	7	Chloroplast
Chr08	*g23996.t1*	*AhSWEET16*	262.00	29.03	9.02	7	Plasma membrane
Chr08	*g24042.t1*	*AhSWEET17*	246.00	27.12	9.30	7	Plasma membrane
Chr08	*g26498.t1*	*AhSWEET18*	301.00	34.32	8.84	7	Plasma membrane
Chr08	*g26499.t1*	*AhSWEET19*	285.00	32.77	9.05	7	Plasma membrane
Chr09	*g27591.t1*	*AhSWEET20*	242.00	26.75	7.63	7	Plasma membrane
Chr10	*g30505.t1*	*AhSWEET21*	292.00	32.46	8.82	7	Plasma membrane
Chr11	*g34946.t1*	*AhSWEET22*	203.00	23.25	8.88	5	Plasma membrane
Chr13	*g40015.t1*	*AhSWEET23*	261.00	29.73	6.99	7	Chloroplast
Chr13	*g42900.t1*	*AhSWEET24*	253.00	27.91	9.37	6	Plasma membrane
Chr13	*g43224.t1*	*AhSWEET25*	249.00	28.29	9.71	7	Plasma membrane
Chr13	*g43226.t1*	*AhSWEET26*	278.00	31.37	9.09	7	Plasma membrane
Chr13	*g44595.t1*	*AhSWEET27*	293.00	32.85	8.49	7	Plasma membrane
Chr14	*g46238.t1*	*AhSWEET28*	240.00	26.36	8.74	7	Chloroplast
Chr14	*g48570.t1*	*AhSWEET29*	261.00	29.73	6.99	7	Chloroplast
Chr14	*g48640.t1*	*AhSWEET30*	274.00	30.31	8.95	7	Plasma membrane
Chr15	*g51208.t1*	*AhSWEET31*	250.00	27.76	9.18	7	Plasma membrane
Chr15	*g51375.t1*	*AhSWEET32*	248.00	27.61	8.72	7	Plasma membrane
Chr16	*g55696.t1*	*AhSWEET33*	244.00	26.62	9.22	7	Plasma membrane
Chr16	*g55713.t1*	*AhSWEET34*	268.00	29.33	9.16	7	Plasma membrane
Chr17	*g59302.t1*	*AhSWEET35*	262.00	28.88	9.01	7	Plasma membrane
Chr17	*g59378.t1*	*AhSWEET36*	246.00	26.95	9.30	7	Plasma membrane
Chr18	*g61739.t1*	*AhSWEET37*	235.00	26.10	7.62	7	Chloroplast
Chr18	*g61743.t1*	*AhSWEET38*	86.00	9.84	4.55	2	Chloroplast
Chr18	*g63200.t1*	*AhSWEET39*	301.00	34.38	8.71	7	Chloroplast
Chr18	*g63201.t1*	*AhSWEET40*	287.00	33.01	9.05	7	Plasma membrane
Chr19	*g64596.t1*	*AhSWEET41*	242.00	26.91	8.21	7	Plasma membrane
Chr19	*g64601.t2*	*AhSWEET42*	90.00	9.83	4.65	2	Plasma membrane
Chr20	*g68189.t1*	*AhSWEET43*	292.00	32.52	8.81	7	Plasma membrane

**Table 2:** Intraspecific gene pairs and Ka/Ks analysis in *Arachis hypogaea*.

Gene Name	Gene Name	Duplication Type	Ka	Ks	Ka/Ks
*AhSWEET1*	*AhSWEET22*	WGD or Segmental	0.000000	0.028507	0.000000
*AhSWEET2*	*AhSWEET24*	WGD or Segmental	0.001771	0.053499	0.033103
*AhSWEET3*	*AhSWEET19*	WGD or Segmental	0.211511	1.215601	0.173997
*AhSWEET3*	*AhSWEET25*	WGD or Segmental	0.000000	0.107755	0.000000
*AhSWEET3*	*AhSWEET40*	WGD or Segmental	0.205203	1.192994	0.172006
*AhSWEET4*	*AhSWEET18*	WGD or Segmental	0.194644	1.546597	0.125853
*AhSWEET4*	*AhSWEET26*	WGD or Segmental	0.000000	0.025887	0.000000
*AhSWEET4*	*AhSWEET39*	WGD or Segmental	0.247602	1.604054	0.154360
*AhSWEET5*	*AhSWEET21*	WGD or Segmental	0.268271	1.278561	0.209823
*AhSWEET5*	*AhSWEET27*	WGD or Segmental	0.011890	0.035691	0.333148
*AhSWEET5*	*AhSWEET43*	WGD or Segmental	0.265571	1.106245	0.240065
*AhSWEET6*	*AhSWEET29*	WGD or Segmental	0.000000	0.000000	NaN
*AhSWEET7*	*AhSWEET30*	WGD or Segmental	0.000000	0.000000	NaN
*AhSWEET8*	*AhSWEET32*	WGD or Segmental	0.000000	0.022265	0.000000
*AhSWEET9*	*AhSWEET31*	WGD or Segmental	0.108679	0.743525	0.146168
*AhSWEET12*	*AhSWEET33*	WGD or Segmental	0.020191	0.016870	1.196820
*AhSWEET13*	*AhSWEET16*	WGD or Segmental	0.182594	0.909559	0.200750
*AhSWEET13*	*AhSWEET34*	WGD or Segmental	0.008242	0.064556	0.127670
*AhSWEET13*	*AhSWEET35*	WGD or Segmental	0.177560	0.930338	0.190855
*AhSWEET14*	*AhSWEET37*	WGD or Segmental	0.033840	0.057062	0.593048
*AhSWEET16*	*AhSWEET34*	WGD or Segmental	0.181272	0.944635	0.191897
*AhSWEET16*	*AhSWEET35*	WGD or Segmental	0.008358	0.027615	0.302657
*AhSWEET17*	*AhSWEET36*	WGD or Segmental	0.008915	0.011594	0.768939
*AhSWEET18*	*AhSWEET25*	WGD or Segmental	0.338281	2.280461	0.148339
*AhSWEET18*	*AhSWEET39*	WGD or Segmental	0.015845	0.051368	0.308461
*AhSWEET19*	*AhSWEET40*	WGD or Segmental	0.004547	0.117449	0.038712
*AhSWEET20*	*AhSWEET41*	WGD or Segmental	0.014840	0.033793	0.439163
*AhSWEET21*	*AhSWEET27*	WGD or Segmental	0.280916	1.303468	0.215514
*AhSWEET21*	*AhSWEET43*	WGD or Segmental	0.006048	0.034264	0.176526
*AhSWEET25*	*AhSWEET39*	WGD or Segmental	0.322324	2.255365	0.142914
*AhSWEET34*	*AhSWEET35*	WGD or Segmental	0.176239	0.986612	0.178630
*AhSWEET1*	*AhSWEET22*	WGD or Segmental	0.000000	0.028507	0.000000
*AhSWEET2*	*AhSWEET24*	WGD or Segmental	0.001771	0.053499	0.033103
*AhSWEET3*	*AhSWEET19*	WGD or Segmental	0.211511	1.215601	0.173997
*AhSWEET3*	*AhSWEET25*	WGD or Segmental	0.000000	0.107755	0.000000
*AhSWEET3*	*AhSWEET40*	WGD or Segmental	0.205203	1.192994	0.172006
*AhSWEET4*	*AhSWEET18*	WGD or Segmental	0.194644	1.546597	0.125853
*AhSWEET4*	*AhSWEET26*	WGD or Segmental	0.000000	0.025887	0.000000
*AhSWEET4*	*AhSWEET39*	WGD or Segmental	0.247602	1.604054	0.154360
*AhSWEET5*	*AhSWEET21*	WGD or Segmental	0.268271	1.278561	0.209823
*AhSWEET5*	*AhSWEET27*	WGD or Segmental	0.011890	0.035691	0.333148
*AhSWEET5*	*AhSWEET43*	WGD or Segmental	0.265571	1.106245	0.240065
*AhSWEET6*	*AhSWEET29*	WGD or Segmental	0.000000	0.000000	NaN
*AhSWEET7*	*AhSWEET30*	WGD or Segmental	0.000000	0.000000	NaN
*AhSWEET8*	*AhSWEET32*	WGD or Segmental	0.000000	0.022265	0.000000
*AhSWEET9*	*AhSWEET31*	WGD or Segmental	0.108679	0.743525	0.146168
*AhSWEET12*	*AhSWEET33*	WGD or Segmental	0.020191	0.016870	1.196820
*AhSWEET13*	*AhSWEET16*	WGD or Segmental	0.182594	0.909559	0.200750
*AhSWEET13*	*AhSWEET34*	WGD or Segmental	0.008242	0.064556	0.127670
*AhSWEET13*	*AhSWEET35*	WGD or Segmental	0.177560	0.930338	0.190855
*AhSWEET14*	*AhSWEET37*	WGD or Segmental	0.033840	0.057062	0.593048
*AhSWEET16*	*AhSWEET34*	WGD or Segmental	0.181272	0.944635	0.191897
*AhSWEET16*	*AhSWEET35*	WGD or Segmental	0.008358	0.027615	0.302657
*AhSWEET17*	*AhSWEET36*	WGD or Segmental	0.008915	0.011594	0.768939
*AhSWEET18*	*AhSWEET25*	WGD or Segmental	0.338281	2.280461	0.148339
*AhSWEET18*	*AhSWEET39*	WGD or Segmental	0.015845	0.051368	0.308461
*AhSWEET19*	*AhSWEET40*	WGD or Segmental	0.004547	0.117449	0.038712
*AhSWEET20*	*AhSWEET41*	WGD or Segmental	0.014840	0.033793	0.439163
*AhSWEET21*	*AhSWEET27*	WGD or Segmental	0.280916	1.303468	0.215514
*AhSWEET21*	*AhSWEET43*	WGD or Segmental	0.006048	0.034264	0.176526
*AhSWEET25*	*AhSWEET39*	WGD or Segmental	0.322324	2.255365	0.142914
*AhSWEET34*	*AhSWEET35*	WGD or Segmental	0.176239	0.986612	0.178630

## Data Availability

The original contributions presented in this study are included in the article. Further inquiries can be directed to the corresponding authors.

## Supplementary Materials

The following supporting information can be downloaded at https://www.mdpi.com/article/10.3390/ijms27156924/s1.
