# Supplementary material for: Genome-Wide Identification of the SWEET Gene Family in Peanut and Prediction of Candidate AhSWEET Genes Associated with Seed Protein Accumulation
Source: Int J Mol Sci. 2026 Aug 1;27(15):6924. doi: 10.3390/ijms27156924 (PMC13467531; doi:10.3390/ijms27156924)
Supplement: Supplementary file 1 [file ijms-27-06924-s001.zip › supplementary figure.pdf]

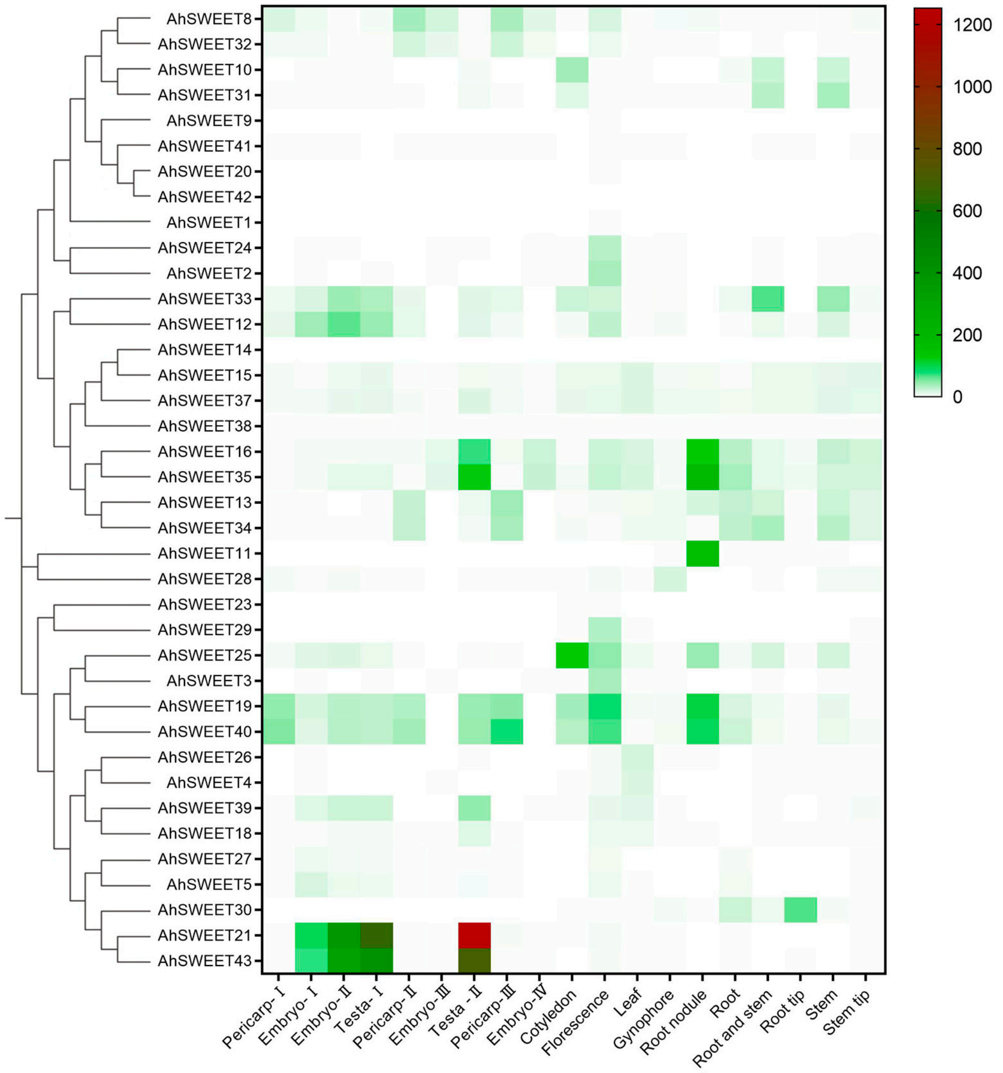

Figure S1. Expression patterns of *AhSWEET* genes in various tissues and stages. The vertical axis indicates the expression patterns of 38 *AhSWEET* genes. The horizontal axis indicates different samples, including Pericarp- Ⅰ（Pod pericarps of 10-20 DAP）、Embryo- Ⅰ（Embryos of 10-20 DAP）、Embryo - Ⅱ（Embryos of 20-30 DAP）、Testa - Ⅰ（Seed testa of 10-30 DAP）、Pericarp- Ⅱ（Pod pericarps of 30-40 DAP）、Embryo -Ⅲ（Embryos of 30-40 DAP）、Testa - Ⅱ（Seed testa of 40-60 DAP）、Pericarp-Ⅲ（Pod pericarps of 50-60 DAP）、Embryo-Ⅳ（Embryos of 40-60 DAP）、Cotyledon、Florescence、Leaf（Leaves mixed）、Gynophore、Root nodule、Root、Root and stem、Root tip、Stem、Stem tip, The gradient colors represent the magnitude of the FPKM values.
